# Suprasellar Mature Cystic Teratoma: An Unusual Location for an Uncommon Tumor

**DOI:** 10.1155/2013/180497

**Published:** 2013-10-02

**Authors:** Raed B. Sweiss, Faris Shweikeh, Fadi B. Sweiss, Stephanie Zyck, Lauren Dalvin, Javed Siddiqi

**Affiliations:** ^1^Department of Neurosurgery, Arrowhead Regional Medical Center, 400 N. Pepper Avenue Colton, CA 92324, USA; ^2^College of Medicine, Northeast Ohio Medical University, 4209 State Route 44, P.O. Box 95, Rootstown, OH 44272, USA; ^3^Department of Neurosurgery, George Washington University, 2150 Pennsylvania Avenue NW, Suite 7-420, Washington, DC 20037, USA

## Abstract

Intracranial germ cell tumors are uncommon and account for only 0.3–3.4% of all intracranial tumors. Teratomas are a subset of these neoplasms, and their finding in brain structures is exceptionally rare, and occurrence within the skull base is quite novel. The authors report the case of a 57-year-old male patient who presented with vision changes, incontinence, ataxia, and altered mental status of 1 week's duration. Imaging revealed a large intrasellar mass with suprasellar extension, involvement of the ventricular system, and marked hydrocephalus with the enlargement of the lateral and third ventricles. The patient underwent a pterional craniotomy/transsylvian approach for resection of the mass. Postoperative histological examination of the resected mass was confirmatory for a mature cystic teratoma. This was followed by radiotherapy, stereotactic radiosurgery, and adjuvant radiotherapy. At the most recent followup, approximately 4 years later, the patient is doing well with improved vision since the operation. This report highlights our experience with a teratoma in a very unusual location, and we review the relevant literature.

## 1. Introduction

Intracranial germ cell tumors of the central nervous system are rare and usually occur in the midline of the brain, particularly at the pineal region and neurohypophysis [1, 2]. While mixed forms are common, multiple types have been described with varying malignant behavior. Specifically, teratomas have been designated as benign tumors that contain representative components of all three germinal layers [3]. 

Teratomas comprise only 0.5% of all intracranial tumors [3]. One study found the age range for central nervous teratomas to be from 16 to 45 years, with the mean age being 15.9 years [4]. Common presenting symptoms of intracranial teratomas may include signs of raised intracranial pressure, visual disturbance, polydipsia, and polyuria [2, 4]. Treatment protocols for malignant intracranial mature nongerminomatous germ cell tumor in adults have been adapted from strategies used in the pediatric population [5]. When imaging studies and CSF analysis are inconclusive, stereotactic or incisional biopsies provide a diagnosis by providing a histopathological sample [5]. Gross total resection may confer a survival benefit and prevent the development of more serious clinical complications [5], with the intracranial location of the tumor deciding the surgical approach [4]. We report on a rare case of a skull-base teratoma with suprasellar extension. 

## 2. Case Presentation

### 2.1. History

The patient is a 57-year-old man who presented in July 2009 with decreased vision bilaterally, left sided facial weakness, ataxia, and short-term memory loss. The patient had also presented previously with a history of seizures, most recently a few months prior to the current presentation. A craniopharyngioma or another parasellar mass was suspected, and an ophthalmology consult was obtained.

### 2.2. Imaging and Laboratory Studies

**Figure 1:**
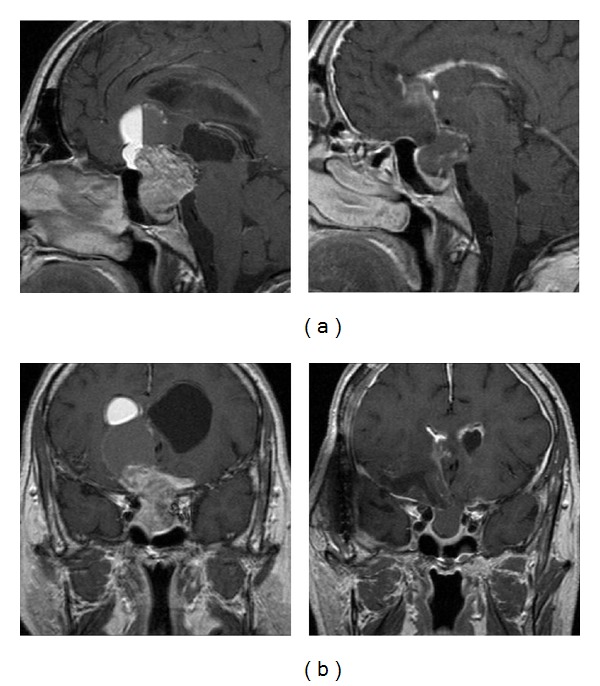
Sagittal T1 with contrast MRIs (a) and coronal T1 with contrast MRIs (b) showing a large sellar mass with significant suprasellar infiltration.

MRI showed a large intrasellar mass with considerable suprasellar extension and chiasmatic compression with the displacement of the inferior frontal lobe. There was also hydrocephalus with gross enlargement of the lateral and third ventricles (Figure 1). A hormone panel showed increased levels of FSH and LH of 46.3 mIU/mL and 12.3 mIU/mL, respectively. Plasma ACTH, IGF-I, growth hormone, prolactin, total cortisol, and testosterone were all within normal limits. CSF analysis identified no malignant cells. 

### 2.3. Operation

The patient was determined to be an excellent candidate for surgery, the only other comorbidity being glaucoma treated with Lumigon. Because of the location of the tumor, it was decided that the patient would undergo a right pterional craniotomy/transsylvian approach for the resection of the tumor and the placement of a ventriculostomy catheter. After placement of a ventriculostomy catheter, the major part of the craniotomy followed. The first line of incision was made in a curvilinear fashion that began at the area 1 centimeter anterior to the tragus of the ear near the root of the zygoma. It curved superiorly up around the temporal bone and parietal bone and then extended toward the midline frontal bone. Dissection proceeded to expose the appropriate amount of cranial anatomy. The tumor was eventually exposed by opening up the sylvian fissure to separate the frontal lobe from the temporal lobe. The tumor was noted to have both cystic and solid components. Fluid aspirated from the cystic component was thick and yellow, with the appearance of oil or cholesterol. There were odd appearing components to the tumor, including what appeared to be yellow clumps of hair embedded within fatty deposits. There were also several firm calcified structures that appeared to be compressing the left optic nerve. The optic nerve itself appeared to be blanched in color without a very good blood supply. There were several firm calcified deposits of tumor that were tightly adhered to the optic chiasm, right carotid artery, and cranial nerve III complex. Though these were tightly adhered and it was decided to be too dangerous to completely resect, the high quality of decompression performed made it so that it was not felt that complete removal of these structures was clinically indicated. 

### 2.4. Pathology

**Figure 2:**
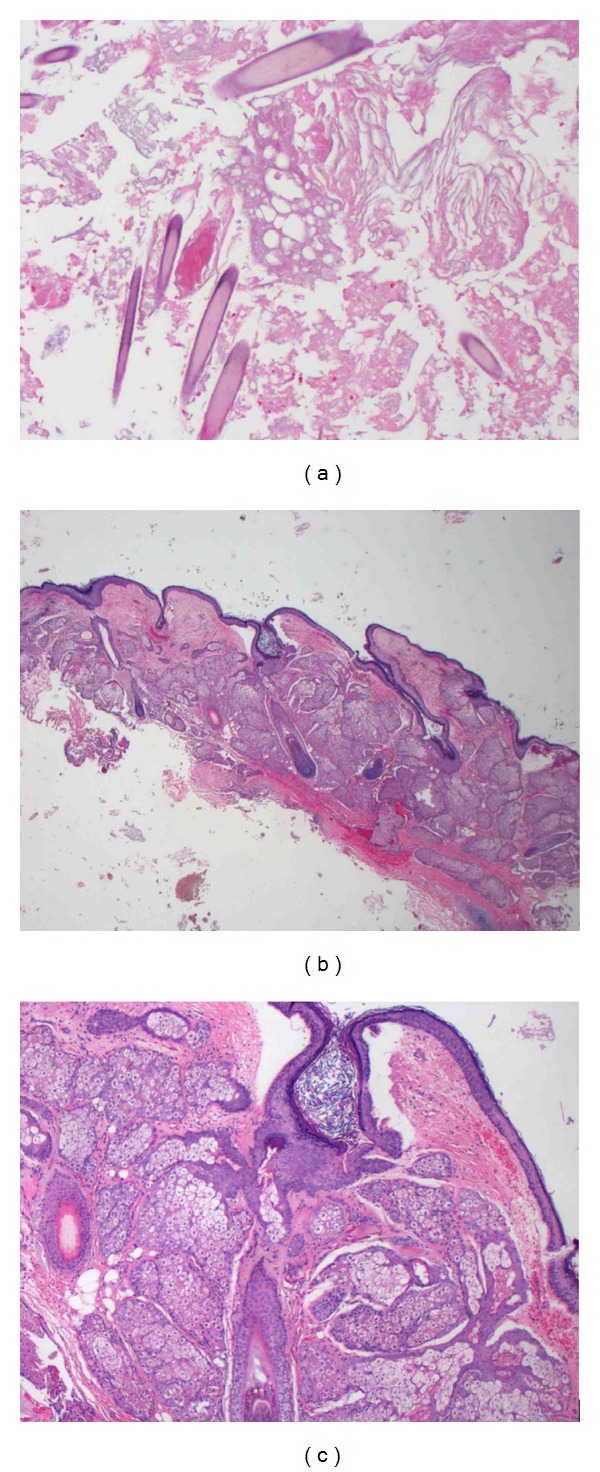
Hematoxylin and eosin (H&E) stained sections of the tumor resection. (a) Cyst contents with hair, keratinous material, and debris. ((b) and (c)) Cyst wall composed of skin and its appendages. Original magnifications: 100x (a), 12.5x (b), and 40x (c).

Specimens taken during the operation were sent to pathology and revealed portions of skin with skin appendages, innumerable hairs, and keratinous debris (Figure 2). Fluid from the cystic component demonstrated a homogenous, yellow gelatinous appearance no gross identifiable solid tissue. Another component of the specimen obtained was a tan-brown, granular appearing tissue with mixtures of curd-like material. No immature tissue component was identified. The mass was identified to be the most consistent with a suprasellar mature cystic teratoma.

### 2.5. Postoperative Course

Upon closure of the procedure, there appeared to be no complications, and postoperative condition was deemed stable. Due to incomplete resection of tumor and risk of recurrence, external beam radiotherapy and eventually stereotactic radiosurgery were used as well as postoperative adjuvant therapy. The patient had a seemingly uncomplicated subsequent recovery and was discharged home. Though over the next month he had a slowly progressive decline in mental status and was admitted for observation. He was discharged soon after following close monitoring and recovery of his status. Since then, he has been watched carefully with frequent clinic appointments and imaging studies. At the most recent followup, approximately 4 years later, he is doing well with significantly improved neurological status and vision.

## 3. Discussion 

Mature teratomas are well-differentiated tumors that are usually lobulated and often firmly adhere to neighboring structures [2]. They contain fully mature tissues of ectodermal, mesodermal, and endodermal origins [2]. Microscopically, they tend to consist of solid and cystic components of squamous epithelium with keratin debris [2]. Components of mature teratomas reported in previous case reports are diverse and include immature mesenchymal tissue, cuboidal and columnar epithelium with goblet cells, islands of immature cartilage, bands of striated and nonstriated muscles, glioneuronal tissue, retinal tissue, and adipose tissue [2]. CT and MRI are helpful to estimate the nature of the lesion and may show components of mixed density that include fat and soft tissue, cartilage, and calcified tissues such as bone and teeth [2]. Here, the authors report the case of a 57-year-old male patient with a skull-base teratoma who had presented with vision changes, incontinence, ataxia, and altered mental status. While CT showed hydrocephalus without other changes from prior films, MRI revealed a large intrasellar mass with suprasellar extension, involvement of the ventricular system, and marked hydrocephalus with the enlargement of the lateral and third ventricles. Postoperative examination of the resected mass revealed a mature cystic teratoma, or dermoid cyst.

Intracranial germ cell tumors are rare comprising about 0.3% to 3.4% of all intracranial tumors [1]. The prevalence is much higher in the earlier decades of life [6], with one peak in the neonatal and infancy period and another peak in children ages of 5–14 [7]. Overall, these tumors appear to be more common in men, with a finding of 79.7% in men versus 20.3% in women reported in one paper [7]. However, despite a clear preponderance of the more common pineal region tumors for male patients, suprasellar (neurohypophysis) region tumors may be more common in women [7]. While germ cell tumors are often mixed in type, five distinct types have been described: germinomas, teratomas, embryonal carcinomas, endodermal sinus tumors (yolk sac tumors), and choriocarcinomas [2, 8–10]. The majority of adult germ cell tumors are germinomas of the testes [8, 11]. 

Teratomas are a type of germ cell tumors containing tissue elements of each of the three germ cell layers: endoderm, mesoderm, and ectoderm [3, 12]. They have been further classified as mature, immature, or malignant [4, 11]. Cranio-spinal axis teratomas are uncommon, and they have been discussed only in case reports and a rare case series [4, 13]. Most intracranial germ cell tumors arise from midline structures, with the pineal gland being the most common, followed by the suprasellar compartment [1]. Germinomas are the most common germ cell tumors in these locations, and it has been suggested that the primary site of suprasellar germinomas is the neurohypophysis [1, 14]. Thus, we find a teratoma in the intra- and suprasellar region of a 57-year-old male to be particularly rare. 

Clinical and radiographic findings can make it difficult to distinguish a mature teratoma from a craniopharyngioma [6]. In this case, craniopharyngioma was suspected prior to histological examination of the original tumor due to the age of the patient, presenting symptoms, and imaging studies. Craniopharyngiomas account for 6% to 10% of all intracranial tumors, making them much more common than teratomas [6, 15]. They often occur in the hypothalamic-pituitary region, and while they are especially common in the first two decades of life, they do have a second prevalence peak in the seventh and eighth decades [6, 15]. Craniopharyngiomas typically show calcification on CT; however, mature teratomas can differentiate into a cartilaginous tissue, which is hyperdense and can mimic calcification. Therefore, our case highlights the importance of obtaining a histological diagnosis to differentiate teratomas from other lesions. 

The typical treatment for mature teratomas is surgical resection, which was successfully done in this case. The deterioration of the patient after a period of improvement in mental status and ambulation may be due to hydrocephalus or due to chemical encephalitis from by-products of the tumor. Mature teratomas are benign and have been reported to have survival rates up to 93% at 10 years [7]. However, a phenomenon of tumor recurrence has been reported, referred to as the growing teratoma syndrome [5, 12]. This refers to a relapse following a partial response to therapy with a tumor that is often cystic and has elements of a mature teratoma, [5, 16]. In this syndrome, the recurrent tumor is presumed to be refractory to chemotherapy or radiation [5]. This syndrome, however, generally refers to those who had a previous malignant tumor. 

## 4. Conclusion

Suprasellar mature teratomas are exceptionally rare. However, because they mimic many other lesions, it is important to recognize this entity in the differential diagnosis and to obtain a thorough histological diagnosis. A multimodal approach for treatment is recommended with complete surgical resection, radiotherapy, and chemotherapy. Future reports on this rare location for a teratoma can help ascertain long-term outcomes.
